# Building internal capacity in pragmatic trials: a workshop for program scientists at the US National Cancer Institute

**DOI:** 10.1186/s13063-019-3934-y

**Published:** 2019-12-27

**Authors:** Wynne E. Norton, Merrick Zwarenstein, Susan Czajkowski, Elisabeth Kato, Ann O’Mara, Nonniekaye Shelburne, David A. Chambers, Kirsty Loudon

**Affiliations:** 1 Division of Cancer Control and Population Sciences, National Cancer Institute, 9609 Medical Center Drive, #3E424, Bethesda, MD 20850 USA; 2 Western University, London, Ontario Canada; 3 Agency for Healthcare Research and Quality, Rockville, MD USA; 4 Independent Contractor, Rockville, MD USA; 5 Edinburgh, Scotland

**Keywords:** Pragmatic trials, Explanatory trials, Cancer prevention and control, Cancer screening, Cancer care delivery research, Cancer behavioral research, Cancer epidemiology, Clinical trials, Real-world evidence

## Abstract

**Background:**

Building capacity in research funding organizations to support the conduct of pragmatic clinical trials is an essential component of advancing biomedical and public health research. To date, efforts to increase the ability to design and carry out pragmatic trials have largely focused on training researchers. To complement these efforts, we developed an interactive workshop tailored to meet the roles and responsibilities of program scientists at the National Cancer Institute—the leading cancer research funding agency in the USA. The objectives of the workshop were to improve the understanding of pragmatic trials and enhance the capacity to distinguish between elements that make a trial more pragmatic or more explanatory among key programmatic staff. To our knowledge, this is the first reported description of such a workshop.

**Main body:**

The workshop was developed to meet the needs of program scientists as researchers and stewards of research funds, which often includes promoting scientific initiatives, advising prospective applicants, collaborating with grantees, and creating training programs. The workshop consisted of presentations from researchers with expertise in the design and interpretation of trials across the explanatory-pragmatic continuum. Presentations were followed by interactive, small-group exercises to solidify participants’ understanding of the purpose and conduct of these trials, which were tailored to attendees’ areas of expertise across the cancer control continuum and designed to reflect their scope of work as program scientists at NCI. A total of 29 program scientists from the Division of Cancer Control and Population Sciences and the Division of Cancer Prevention participated; 19 completed a post-workshop evaluation. Attendees were very enthusiastic about the workshop: they reported improved knowledge, significant relevance of the material to their work, and increased interest in pragmatic trials across the cancer control continuum.

**Conclusion:**

Training program scientists at major biomedical research agencies who are responsible for developing funding opportunities and advising grantees is essential for increasing the quality and quantity of pragmatic trials. Together with workshops for other target audiences (e.g., academic researchers), this approach has the potential to shape the future of pragmatic trials and continue to generate more and better actionable evidence to guide decisions that are of critical importance to health care practitioners, policymakers, and patients.

## Background

As first articulated by Schwartz and Lellouch [1] , clinical trials can be conceptualized across a continuum from explanatory to pragmatic, with the former assessing if a practice, intervention, program, or treatment could work under ideal circumstances and the latter determining if it could work in routine practices. Both explanatory and pragmatic trials—and those that fall between the ends of the continuum—are essential for advancing research and practice in health care and public health. Pragmatic trials in particular are necessary for developing and testing interventions using the settings, resources, patients, and approaches to which they will ultimately be implemented, thus ensuring that research funds have the greatest potential to impact patient and population health. To this end, the United States (US) National Cancer Institute (NCI) leadership [2] has included the support and conduct of pragmatic trials as part of its recommendations for modernizing the clinical trial enterprise.

In recent years, there has been a significant increase in the number of pragmatic trials across a range of health areas, intervention types, delivery settings, and patient populations. Although the conceptualization of trials along the continuum has advanced over the years, there remains great variability in how pragmatic trials are defined, described, operationalized, and labeled. A recent study by Dal-Ré and colleagues [3] found that 36% (32) of self-labeled pragmatic trials (*N* = 89) more accurately reflected characteristics of explanatory trials (e.g., placebo-controlled, single-center). Similarly, in a review of recently published trials identified in the article title as pragmatic, 33 (45%) provided no justification in text for why their trial was pragmatic [4]. Although there is no single “correct” way to describe trials along the continuum, significant misclassification may provide misleading information to health care practitioners, policymakers, advocates, and patients about the applicability of the trial results to everyday settings and populations.

To remediate some of the confusion, and to help build capacity in pragmatic trials, workshops, short courses, tools, and online resources have been developed for researchers interested in conducting such trials. For example, in the USA, the National Institutes of Health (NIH) Health Care Systems Research Collaboratory has an online Living Textbook on pragmatic trials, which includes self-paced learning modules, factsheets, web-based presentations, podcasts, and starter kits on pragmatic trials geared toward investigators and research teams [5]. The Health Care Systems Research Collaboratory also hosts in-person training workshops on pragmatic trials for researchers with workshop materials available online. The ACCORDS Dissemination and Implementation Science Program of the Anschutz Medical Campus, University of Colorado, Denver, hosts an interactive, user-friendly training eBook for researchers, *Pragmatic Trials: A Workshop Handbook* [6]. The edX platform hosts a massive open online course, *Pragmatic Randomized Controlled Trials in Health Care* [7]. *PragMagic* is an online pragmatic trial resource designed to aid in the design, conduct, and evaluation of pragmatic trials, and includes the *PragMagic Tool* [8], a decision support tool for trial design.

One of the most commonly used resources is the Pragmatic Explanatory Continuum Indicator Summary (PRECIS) tool. First published in 2009, it was developed to aid researchers in considering the overall purpose and intent of their proposed trial, and the applicability of trial results to real-world settings [9]. Revised in 2015 [10], the PRECIS-2 tool consists of 9 key domains (i.e., eligibility, recruitment, setting, organization, flexibility [delivery], flexibility [adherence], follow-up, primary outcome, and primary analysis) that distinguish trials along the continuum from explanatory to pragmatic. Each domain represents a component of the trial that can make it more explanatory or more pragmatic. Domains are scored through discussion during the planning phase of the trial among the research team, with a score of 1 reflecting a more explanatory trial and a score of 5 reflecting a more pragmatic trial; scores are visually represented on a PRECIS-2 wheel, where wheels close to the hub reflect more explanatory trials and wheels close to the rim reflect more pragmatic trials. The PRECIS-2 tool is considered by many to be the gold standard for conceptualizing trials along the continuum. To date, and to the best of our knowledge, it is also the only tool that has demonstrated good inter-rater reliability and discriminant validity [11]. PRECIS-2 is often included as part of workshops and trainings on pragmatic trials. To facilitate trialists, an interactive web-based platform assists registered users in completing a PRECIS-2 wheel specific to their trial [12]. To date, over 700 international researchers have used this website software.

Despite the increasing number of resources for building capacity among investigators in pragmatic trials, few—if any—opportunities are designed to meet the unique needs of funding agency staff in medical research agencies. Across the NIH, program scientists are responsible for many scientific and administrative aspects of research. At the NCI, 1 of 27 institutes, centers, and offices of the US NIH, a leading medical research and funding agency, responsibilities of program scientists include (but are not limited to) the following: (1) assuming scientific leadership by defining short- and long-range goals for research programs, (2) stimulating interest in projects and special activities through communication with the scientific community, (3) identifying areas warranting either increased or decreased funding emphasis, (4) developing funding opportunity announcements to solicit applications from the scientific community, (5) consulting and advising grantees during the preparation of applications, and (6) managing funded research projects and research training programs [13]. The roles and responsibilities of program scientists differ from those of investigators and, accordingly, require different skill sets, training, and expertise, as their main responsibilities are tied to the goals of the funding agency in supporting and growing targeted areas of science rather than conducting independent research. Thus, program scientists are in a unique role of interacting directly with the research community while also creating research opportunities in targeted health areas.

This commentary describes an interactive, tailored workshop on pragmatic trials to meet the needs of program scientists and developed to complement existing training programs for other target audiences (e.g., academic researchers). The workshop focused on a specific health area—cancer—and included 29 program scientists working in 2 NCI divisions (i.e., Division of Cancer Control and Population Sciences and Division of Cancer Prevention), both divisions of which are heavily involved in developing and testing interventions across the cancer control continuum (i.e., etiology, prevention, detection, diagnosis, treatment, and survivorship) in real-world public health and cancer care delivery settings.

### Pragmatic trials across the cancer continuum: an interactive workshop

The 6-h, in-person workshop consisted of a combination of didactic presentations from experts in the field and small-group activities customized to help program scientists with their everyday responsibilities. A workshop planning committee (SC, EK, AOM, NS, WN) was formed and included representatives from across the cancer control continuum in the Division of Cancer Control and Population Sciences and the Division of Cancer Prevention. The planning committee was responsible for developing the objectives, content, and structure of the workshop didactic portion and small-group activities (e.g., case studies, facilitator guides, group composition). Consistent with best practices in workshop development [14, 15], a pre-workshop online survey assessed participants’ general understanding of pragmatic trials, which in turn guided workshop content, scope, and selection of supplementary materials (e.g., list of key references and websites, PRECIS-2 toolkit, frequently asked questions document, and copies of seminal articles). The 1-day workshop was held in October 2018 at NCI in Rockville, MD, USA. A detailed copy of the workshop agenda can be found in Table 1.

**Table 1:** Agenda for pragmatic trials across the cancer continuum interactive workshop

Workshop session	Format and brief description
Background and purpose of the workshop	Brief presentation orienting attendees to the overall purpose of the workshop, its applicability to advancing research along the cancer control continuum, and how trials along the explanatory-pragmatic continuum fit within the mission statements of the Division of Cancer Control and Population Sciences and the Division of Cancer Prevention at the National Cancer Institute.
Introduction to explanatory and pragmatic trials	Expert presentation providing a brief overview of randomized controlled trials, detailed explanation of how trials differ in attitude and purpose along the explanatory-pragmatic continuum, and illustrative examples of explanatory and pragmatic trials in health research.
PRECIS-2: a tool for planning trials	Expert presentation describing PRECIS-2, including why and how it was developed, walk-through of visual representation (PRECIS-2 wheel), steps for how to use the PRECIS-2 tool, explanation of the nine domains for scoring trials along the explanatory-pragmatic continuum with illustrative examples in cancer research, and review of key resources.
Small-group activity #1	Small-group interactive session with facilitated discussion. Groups were provided with a copy of one of three cancer-focused trials from the peer-reviewed literature. One trial was more pragmatic, one trial was more explanatory, and one trial had aspects of both. Two groups were assigned the same trial. Through discussions, each group scored the PRECIS-2 domains for one trial and identified corresponding text that reflected their score.
Full-group report-out #1 and discussion	Full-group report-out with facilitated discussion. Each small group presented domain scores and corresponding text for their assigned trial to the full group of attendees. Divergent scores between the two groups assigned to the same trial were discussed, as were domains that were particularly challenging to rate.
Small-group activity #2	Small-group interactive session with facilitated discussion. Each group received the same three documents: copy of article of explanatory trial, copy of pre-populated PRECIS-2 scoring table, and copy of blank PRECIS-2 scoring table. Participants were asked to provide suggestions for how to make the trial more pragmatic.
Full-group report-out #2 and discussion	Full-group report-out with facilitated discussion. Each group presented suggestions for how to make the trial more pragmatic for each of the nine PRECIS-2 domains. Experts moderated the discussion.
Open discussion and workshop conclusion	Open Q&A and discussion format with all participants and experts.

The PRECIS-2 tool and supplemental documents (e.g., wheel, domain descriptions, examples) can be found at https://www.precis-2.org/

### Presentations

The workshop began with an overview of the purpose and structure of the workshop (WN), followed by two 45-min expert presentations (MZ, KL), each including group discussion. The presentations introduced attendees to the concepts of explanatory and pragmatic trials and reviewed the PRECIS-2 tool for operationalizing trials along the continuum. The first expert discussed the strengths of randomized controlled trials (RCTs) in health [16], reviewed several limitations of RCTs (e.g., poor generalizability, applicability, and external validity [17, 18]), and introduced the conceptualization of RCTs along a multiaxial explanatory-pragmatic continuum [1, 19, 20]. The second presentation reviewed the most widely used tool, PRECIS-2, for operationalizing trials along the continuum during the design phase of the study [10]. Examples from cancer prevention and control trials were used to illustrate scores for each of the nine domains in the PRECIS-2 tool. Following the plenary component, participants attended two interactive sessions to enhance the application of knowledge.

### Activity #1

The objective of the first group activity was to enhance attendees’ understanding of explanatory and pragmatic trials in cancer prevention and control, reflecting the range of trials that program scientists manage and advise on a daily basis. Attendees were divided into six groups consisting of a mix of individuals from epidemiology, behavioral science, health care delivery, implementation science, and symptom management/palliative care program areas. Each group was assigned one of three published articles on a cancer-focused trial: one trial that was more pragmatic, one trial that was more explanatory, and one trial that had aspects of both. The participating program scientists deliberated in small groups and (1) scored their trial using the PRECIS-2 tool, (2) identified relevant text excerpts reflecting their domain score, and (3) discussed their score for each domain. The PRECIS-2 toolkit, which includes the PRECIS-2 wheel, domain descriptions, and examples from published trials, all of which are downloadable on the PRECIS-2 website [12], served as template documents for the activity. Facilitators guided the discussion and asked probing questions, as needed, using a standardized “answer” sheet—a version of the PRECIS-2 tool that had been completed by the steering committee and consultants for each trial before the workshop. A blank PRECIS-2 wheel on a flip chart was available for each group to record their domain ratings. All attendees reconvened in plenary to discuss the groups’ consensus on the trial ratings and rationale. Two flip charts (one per group) were posted side by side for each of the three trials to visualize agreements and discrepancies in domain scores. Divergent scores and corresponding text were discussed, and domains that were particularly challenging to rate received extra attention. Discussion of the discrepancies provided an opportunity to emphasize the importance of a team-based approach for scoring domains as well as highlighting the reality that some variability in scores is expected, as there are no objectively correct or incorrect ratings.

### Activity #2

The objective of the second small-group activity was to facilitate the program scientists’ ability to characterize the components of trials that would reflect a more pragmatic vs. a more explanatory design and thus be able to guide investigators should they be aiming for the former. This activity was designed to mirror the program scientists’ role in providing technical assistance to prospective grantees during the pre-submission process (e.g., feedback on overall study aims), which may include a discussion of research approach (e.g., designs, methods, theories, measures, outcomes), innovation, significance of the proposed study, and fit with overall program goals. To this end, participants across all groups received the same three documents: (1) a copy of an article of a relatively explanatory trial comparing modalities to increase colorectal cancer screening, (2) a copy of a PRECIS-2 scoring table that was pre-populated with domain scores and extracted text from that trial justifying the score, and (3) a copy of a blank PRECIS-2 scoring table. Again, the PRECIS-2 toolkit served as a template for the documents. With guidance from the facilitator (each of whom had a pre-scored version of the PRECIS-2 table), the groups were asked to provide suggestions for how to make the trial more pragmatic. As with the first activity, all participants reconvened and presented suggestions for how to make the trial more pragmatic, which led to an engaging discussion about the characteristics of pragmatic vs. explanatory trials, moderated by the two experts.

### Evaluation

A paper-based questionnaire was administered at the end of the workshop and collected by non-NCI staff who assisted with the workshop logistics and preparation. The evaluation consisted of six items to assess the relevance and effectiveness of the workshop (e.g., “Activities were relevant to my professional role,” “Workshop was a good use of my time,” and “Presentations increased my understanding of the explanatory-pragmatic continuum,” all scored from 1 = strongly disagree to 5 = strongly agree). Participants were asked to indicate which, if any, resources or activities they would like as a follow-up to the workshop (e.g., interest group; working group to develop sample text on pragmatic trials for relevant funding opportunity announcements). Finally, attendees were asked to indicate what they liked most about the workshop, suggestions for improvement, and any additional feedback or comments (open-ended response option). Participants were given the option of providing their name at the end of the survey.

Nineteen of the 29 attendees (66%) completed the post-workshop evaluation. Overall, participants rated the workshop very positively. Most attendees were interested in follow-up activities to help educate researchers in pragmatic trials (e.g., hosting webinars, *n* = 13; providing training opportunities, *n* = 13), with relatively fewer interested in internal activities (e.g., NCI interest group, *n* = 11; trans-NIH interest group, *n* = 8). Most respondents (*n* = 14) reported the interactive exercises to be the most beneficial. As one respondent noted, “Activities were extremely helpful in contextualizing the material.” Another individual stated, “I really liked that we were able to apply what we learned in the slides to real-world examples.” A range of improvements were suggested, including additional time for the activities and group discussions, access to reading materials beforehand, and a greater variety of cancer-focused interventions as examples.

## Conclusions

Building interest in and understanding of pragmatic trials is essential for expediting the application of trial results to usual practice settings and patient populations. To complement the existing trainings for other target audiences (e.g., academic researchers), we developed an interactive workshop tailored specifically to meet the needs of program scientists in their role at a major biomedical research funding agency. Feedback on the workshop was overwhelmingly positive. We anticipate that attendees will be more likely to incorporate knowledge gained from the workshop into a range of professional activities in the future.

This workshop may serve as a template for research funding agencies to adapt and use to build internal capacity in supporting the conduct of pragmatic trials, and to complement existing trainings for other target audiences. Collectively, such opportunities are likely to increase support for the conduct of pragmatic trials among funding agencies and researchers alike. Increasing the number and quality of pragmatic trials can help ensure that research investments directly impact health care practitioners, policymakers, and patients.

## Data Availability

The evaluation data reported herein are not publicly available as individual privacy could be compromised.

## Abbreviations

NCI: National Cancer Institute
NIH: National Institutes of Health
PRECIS-2 (version 2): Pragmatic-Explanatory Continuum Indicator Summary-2
RCT: Randomized controlled trial
